# High-Throughput Optofluidic Acquisition of Microdroplets in Microfluidic Systems

**DOI:** 10.3390/mi9040183

**Published:** 2018-04-14

**Authors:** Zain Hayat, Abdel I. El Abed

**Affiliations:** Laboratoire de Photonique Quantique et Moléculaire, UMR 8537, Ecole Normale Supérieure Paris Saclay, CentraleSupélec, CNRS, Université Paris-Saclay, 61 avenue du Président Wilson, 94235 Cachan, France; zain.hayat@ens-paris-saclay.fr

**Keywords:** microfluidics, droplets, optofluidics

## Abstract

Droplet optofluidics technology aims at manipulating the tiny volume of fluids confined in micro-droplets with light, while exploiting their interaction to create “digital” micro-systems with highly significant scientific and technological interests. Manipulating droplets with light is particularly attractive since the latter provides wavelength and intensity tunability, as well as high temporal and spatial resolution. In this review study, we focus mainly on recent methods developed in order to monitor real-time analysis of droplet size and size distribution, active merging of microdroplets using light, or to use microdroplets as optical probes.

## 1. Introduction

The advent of segmented phase flow in microfluidic systems nearly two decades ago gave rise to the development of an important sub-field of microfluidics known as droplet microfluidics [1,2]. This technology allows for the fabrication and manipulation of millions of highly monodisperse microdroplets, each of which may be regarded as an independent micro-reactor [3,4,5,6,7,8,9,10,11]. The combination of the high flexibility of microfluidics and the compartmentalization of reagents in droplets at high throughput provides powerful automated tools for optimizing chemical synthesis and the development of rapid and low-cost digital assays. Droplets content can be incubated, split, merged, analyzed or sorted at kHz rates, which has proven, for instance, to be a powerful tool to find mutants of genes among a very large population of wild genes [12,13,14,15,16,17,18].

Optofluidics is another fast-growing research field dedicated to the study of the interaction of light with discrete volumes of liquids in microfluidic systems. One may remark that, besides the present themed collection dedicated to optofluidics, two previous themed collections have recently been published in *Lab on a Chip* (in 2013 and 2016) [19,20,21,22,23,24,25,26,27,28,29,30,31,32], as well as a series of international conferences held annually in China since 2011. The combination of droplet microfluidics and optofluidics, coined hereafter as droplet optofluidics, offers many prospects spanning many academic and industrial fields in biology, chemistry, physics, material, and interface sciences [33,34,35,36,37,38,39,40,41,42,43,44,45,46,47,48,49,50,51]. For instance, it enabled the identification of very rare gene sequences [12,13,14,15,16,17,18], screening of cells or bacteria [52], membrane proteins inhibitors screening [53], coupled optical lab-on-chip platform with small angle X-ray scattering (SAXS) [54], and engineering microparticles for photonics applications [55,56], on-chip multiphasic tunable grating [57], reconfigurable droplet grating [58], fluidic Michelson interferometer [59], droplet grating with polydimethylsiloxane (PDMS) air-lens waveguide setup [60], 3D and 4D optically fabricated complex geometries [61,62], reconfigurable compound micro-lenses [63], and countless other possibilities. Previous examples may be considered to be the state-of-the-art, and the domain is flourishing day-by-day to new trends.

Droplet size and droplet size distribution are among the most relevant and challenging characteristics of droplets, and can affect their use for highly quantitative analysis and biological assays. Various methods have been developed in order to monitor the size and size distribution of large populations of droplets in real-time. Some methods employ expensive equipment, such as dynamic light scattering [40], automated scanning electron microscopy [41], acoustic attenuation spectroscopy [42,43], and capillary hydrodynamics [44,45]. New methods based on the state-of-the-art microscopy have also been recently reported in the literature, such as image processing [46,64], real-time on-chip imaging and droplet-sorting systems based on real shape recognition methods [65,66], a coupled bright-field and fluorescence multi-imaging flow cytometer [67], and advanced digital acquisition [47]. In this review, we will focus on the developed optofluidics methods for monitoring and acquiring droplets size and size distribution. We will also tackle some of the engineering as well as crucial aspects of optofluidics methods for droplet manipulation.

## 2. Basics of Droplet Microfluidics Technology

Several materials have been used for the fabrication of microfluidic systems, each of which has its advantages and drawbacks. Silicon was first used for the development of microfluidic chips [68,69]. The reason for selecting silicon is evident from its inert behavior in regards to a wide range of chemical compounds. Being opaque (a major drawback), silicon was soon replaced by glass, which is not only chemically inert, but also transparent [70,71]. The development of glass microfluidic systems requires complicated design protocols. It is worth noting that many materials may be used for microfluidic systems, including thermoplastic [72,73], ceramic laminated sheets [74], thermoset polyester [75], polystyrene (PS) [76,77], poly-methyl-methacrylate (PMMA) [78,79] and polycarbonate (PC) [80,81]. However, polydimethylsiloxane (PDMS) is one of the most widely used polymers for the fabrication of reliable and cost-effective microfluidic devices [82,83]. Among the numerous advantages of PDMS to microfluidics, one may note for instance its excellent optical transparency, easy processing, low cost, mechanical flexibility, long-term stability, biocompatibility and low toxicity, chemical inertness, etc. Nevertheless, PDMS has one main drawback: its propensity for swelling in the presence of low molecular weight organic solvents such as acetone, ethanol, chloroform, etc., which may impede some applications requiring the use of low molecular weight organic solvents, for instance.

**Figure 1:**
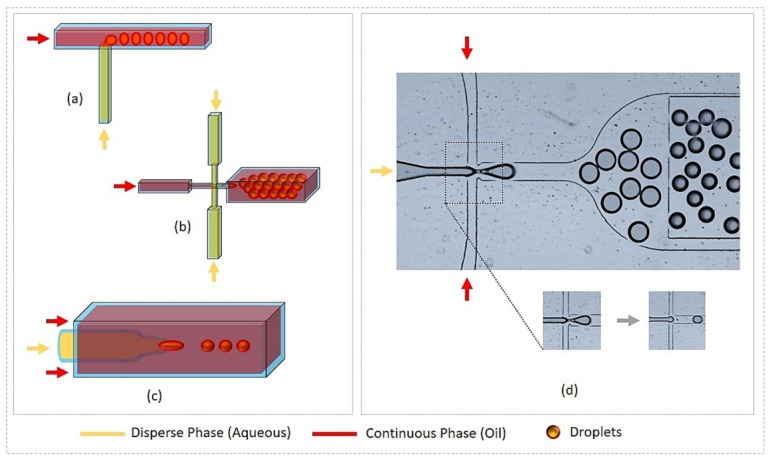
Droplet generation by microfluidic systems. (**a**) T-junction; (**b**) Flow-focusing; (**c**) Co-flow (glass capillary); (**d**) Droplet generation by flow focusing device (use of fluorinated oil with stabilizing agent and disperse phase as water solution of dye), device also includes on-chip storage pool for droplet collection and operation region.

In order to generate highly monodisperse droplets, different designs have been developed. Some of them were developed at the very beginning of droplet microfluidics technology, such as T-junction [84,85,86,87], flow-focusing [32,37,39,47,65,88,89,90,91], co-flow [92,93,94,95], or glass capillary droplet generator [70,71,96,97]. New commercial platforms using micro-pipetting [98,99] are also available; for example, rotAXYS ^®^ (Cetoni) and Dropix^®^ (Dolomite). Nevertheless, T-junction and flow-focusing are the most commonly used droplet generators. They enable a continuous carrier oil flow to periodically slice, at high throughput, a second phase flow (dispersed phase) into tiny droplets at the nozzle region of the microfluidic device (see Figure 1d) [100]. The success of such an operation and the features of the fabricated droplets depend on parameters like the non-miscibility of the two fluids, flow rates (or flow velocity, *u*) of the fluids, interfacial tension (γ) of the two fluids, viscosity (η) of the carrier oil phase, the microchip nozzle size, etc. The mechanism lying behind the formation of highly monodisperse droplets in microfluidic devices is governed by a subtle balance between capillary forces minimizing the interfacial energy between the two fluids (through the formation of spherical droplets) and viscous forces acting on the dispersed phase during the deformation of the interface and the formation of the droplets. The result of a such balance may be evaluated using the dimensionless capillary number $C_{a}$, which is defined as the ratio between the viscous forces and the capillary forces, or $C_{a}$~$\frac{\eta u}{\gamma }$. Depending on the value of the capillary number, three different flow regimes are observed: dripping, jetting, and parallel flow. The first regime, where monodisperse periodic microdrops are produced near the microfluidic device nozzle, is obtained when capillary forces dominate viscous forces (i.e., when $C_{a}<<1$). The second regime is observed when viscous forces become comparable to capillary forces ($C_{a}$~1). This regime is characterized by a long undulating jet, which breaks far downstream from the nozzle into polydisperse droplets. The third regime corresponds to the case where the two fluids flow continuously side by side, and is observed for high flow rates and/or highly viscous fluids (i.e., when $C_{a}>>1$) [101,102,103,104].

To serve successfully as independent microreactors, droplets should obey at least the following specifications: (i) high monodispersity; (ii) long-term stability; (iii) absence of cross-contamination between droplets; and (iv) for biomedical applications, their content and the surrounding medium should be biocompatible. All these specifications can be achieved by using a perfluorinated oil as a carrier fluid and a perfluorinated surfactant as a stabilizing droplet agent [105,106,107,108]. Indeed, perfluorinated compounds are chemically inert and mix neither with aqueous solutions nor with organic solvent solutions.

Perfluorinated oils possess two other important features: (i) PDMS-based microfluidic devices swell much less in their presence than in the presence of hydrocarbon oils, (ii) perfluorinated oils absorb large quantities of oxygen and carbon dioxide, which appears to be a very important feature for the encapsulation of cells and other living organisms in droplets. However, particular care should be taken regarding the used surfactant concentration range in order to avoid mass exchange between droplets, which was shown to occur easily at concentrations above the cmc (critical micellar concentration) [109,110,111,112,113]. Many suitable perfluorinated oils (e.g., HFE 7500 or FC 40) are commercially available (3M company, St Paul, MN, USA). Perfluorinated surfactants can either be purchased from RainDance Technologies (Billerica, MA , USA) or Dolomite (Royston, UK), or home-made according to a reaction scheme initially developed by Holtz et al. [105]. The chemical synthesis is based on the condensation of Krytox FSH-157^TM^ (Kry, a perfluoro-polyether (PFPE) carboxylate from DuPont^®^ which acts as an oil-philic moiety) and polyether derivatives like polyethylene glycol (PEG) or Jeffamine^®^ polyetheramine (Huntsman corp.), playing the role of hydrophilic moieties [32,105,107,108]. Commonly used surfactants include the triblock kry-PEG-kry (or PEG-kry2) [105] or the diblock Kry-Jeffa (a contraction of Krytox and Jeffamine^®^) [107]. One may note that Krytox may also be used as a surfactant in many experiments where biocompatibility is not required. Nevertheless, the negative charge of the Krytox carboxylate group interacts with oppositely charged biomolecules, which may cause the encapsulated biomacromolecules in droplets to lose their activity and aggregate at the droplet interface. Biocompatible perfluorinated surfactants have also been recently reported, such as LPG-Kry2, where the hydrophilic moiety is a linear poly-glycerol (LPG) derivative [108].

## 3. Real-Time Fluorescence Measurements of Droplet Size and Size Distribution

**Figure 2:**
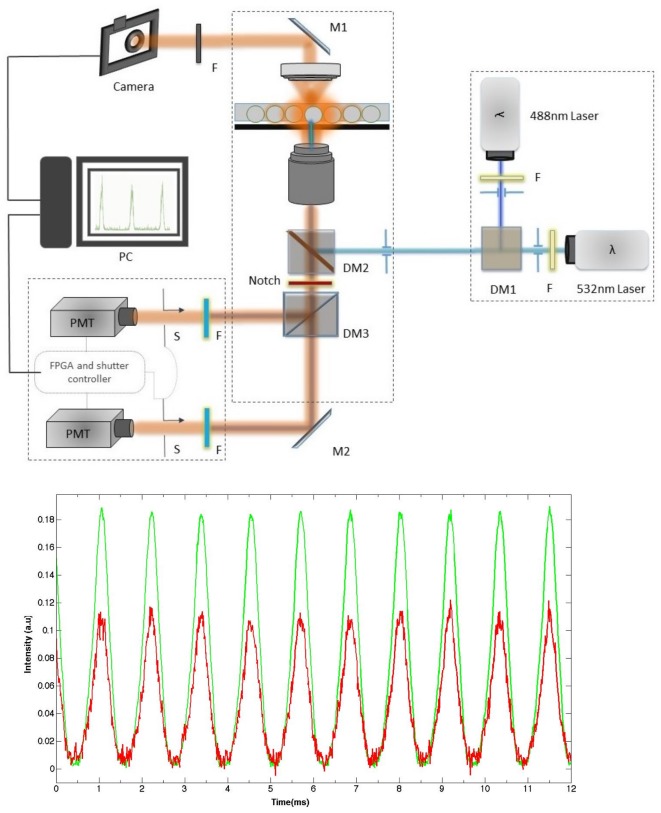
(**top**) A typical dual channel microfluidic droplet monitoring setup, consisting of two laser sources with each laser band limited by a bandpass filter. The two components of the fluorescence signals emitted by the two different dyes are separated, filtered, and collected on two different photo-multiplier tubes (PMTs). (**bottom**) Typical recorded fluorescence intensity versus time emitted by flowing droplets containing both fluorescein and rhodamine dyes at different concentrations. DM: dichroic mirror.

For biomedical applications, fluorescence detection is one of the most popular methods. It allows for real-time monitoring of droplet generation rate and droplet analysis. One or more fluorescent probes are generally used. In the case of dual fluorescence acquisition, for instance, one probe is used for the detection of the droplet content or for the detection of a specific biomarker, while the second probe is used for the detection of a second biomarker of interest. A standard dual fluorescence acquisition setup is depicted in Figure 2. It includes two laser sources optimized for the absorption of the two fluorophores. Laser incident beams are combined by means of a first dichroic mirror (DM1) and then directed towards microdroplets in the microfluidic channel by a second dichroic mirror (DM2). The focused band limited light is targeted towards the droplets and recollected by the microscope objective, which is then transmitted through another set of band-limited filters to two photo-multiplier tubes (PMTs). The signal output from PMTs are then collected at high acquisition rates (~100 kHz) using a data acquisition card (DAQ, National Instruments) and analyzed using FPGA (Field-Programmable Gate Array, Labview, National Instruments) module scripts, which allows for the identification of droplets by the modulation of fluorescence versus time.

Figure 2 shows a typical fluorescence intensity real-time recording from droplets flowing in a 30 μm × 30 μm wide microchannel. Each pulse corresponds to the passage of a single droplet, each of which contains two fluorescent dyes (fluorescein and rhodamine in this experiment), which are excited by two continuous wave (CW) lasers at 488 nm and 532 nm. The duration of each pulse corresponds to the residence time (τ) of a single droplet under the illuminated area (lasers footprint) of the microfluidic channel. Measuring the τ value may allow in principle for the determination of the size of the corresponding droplet, provided that the droplet velocity is known. However, if in a single phase flow the mean velocity value, $\bar{u}$, can be easily determined from the flow features—namely the flow rate *q* and the cross-sectional area *S* of the microfluidic channel ($\bar{u}=\frac{q}{S}$). This task proves to be cumbersome in the case of a flow laden with deformable microdroplets and more particularly in the presence of large droplets. In this case, the flow is strongly modified due to the formation of a thin lubrication oil film between the droplet interface and the microchannel walls. The presence of such a film has a direct effect on the velocity of the droplets and makes the flow pattern complex and difficult to analyze [114,115,116,117,118,119,120,121,122,123,124,125,126]. It has been shown, for instance, that depending on the geometry of the channel, the lubrication film may move either backwards in the case of a cylindrical channel (in regards to a reference frame attached to the droplet) or forward in the case of rectangular or square channels. In the last case, one should take account of the presence in the flow of the continuous oil phase along the gutters of the rectangular or square channels [3,5,127,128].

The determination of the size of droplets becomes straightforward if one accounts for the droplets’ generation frequency *f* and the flow rate of the dispersed phase $Q_{d}$, as we demonstrate hereafter in the case of small spherical droplets. Let $D_{dr}$ and $V_{dr}=\frac{\pi }{6}D_{dr}^{3}$ be their diameter and individual volume, respectively. Since the volume $V_{dr}$ is injected in the microchannel during a period of time $T=\frac{1}{f}$ separating the generation of two successive droplets, one deduces easily $D_{dr}$ according to $D_{dr}={\left(\frac{6}{\pi }\frac{Q_{dr}}{f}\right)}^{\frac{1}{3}}$. We deduce for instance in the case where *f* = 862 Hz and $Q_{d}$ = 25 μL/h (results shown in Figure 2), a droplet size $D_{dr}$ = 24.8 μm, which compares very well with the mean droplet size measured directly using droplet image analysis from droplets collected in a dedicated microfluidic observation chamber—that is , 26 μm (results not shown).

**Figure 3:**
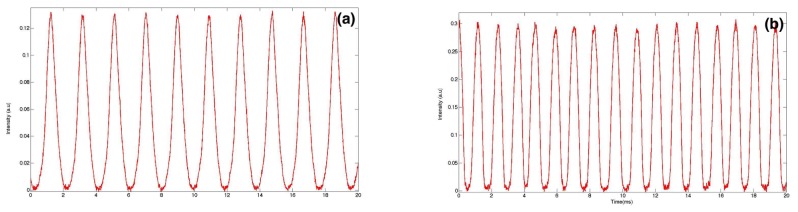
Fluorescence signal extracts from setup (**a**) without surfactant; (**b**) with surfactant.

Real-time fluorescence measurements can also give a valuable insight into the effect of surface tension value on the droplet size, as illustrated in Figure 3. This figure shows fluorescence intensity recordings versus time from two types of droplets produced with two different values of interfacial tension (all other parameters were kept constant; e.g., flow rate of the dispersed phase was $Q_{d}=30\;\mu$L/h). One finds that the droplet size decreased from 31.3μm to 26.7μm as the surface tension decreased from $\gamma_{1}=18$ mN/m (Figure 3a) and $\gamma_{2}=13$ mN/m (Figure 3b), respectively, in agreement with a model suggested earlier by Nguyen et al. [129], where the droplet size was shown to vary as the square root of the interfacial tension: $D_{dr}\propto \sqrt{\gamma }$. The observed change in droplet size can be understood in terms of lowering of the energy cost to build the interface between the two non-miscible phases when the interfacial energy decreases.

## 4. Highly Sensitive Analysis of Droplet Content and Droplet Interface

**Figure 4:**
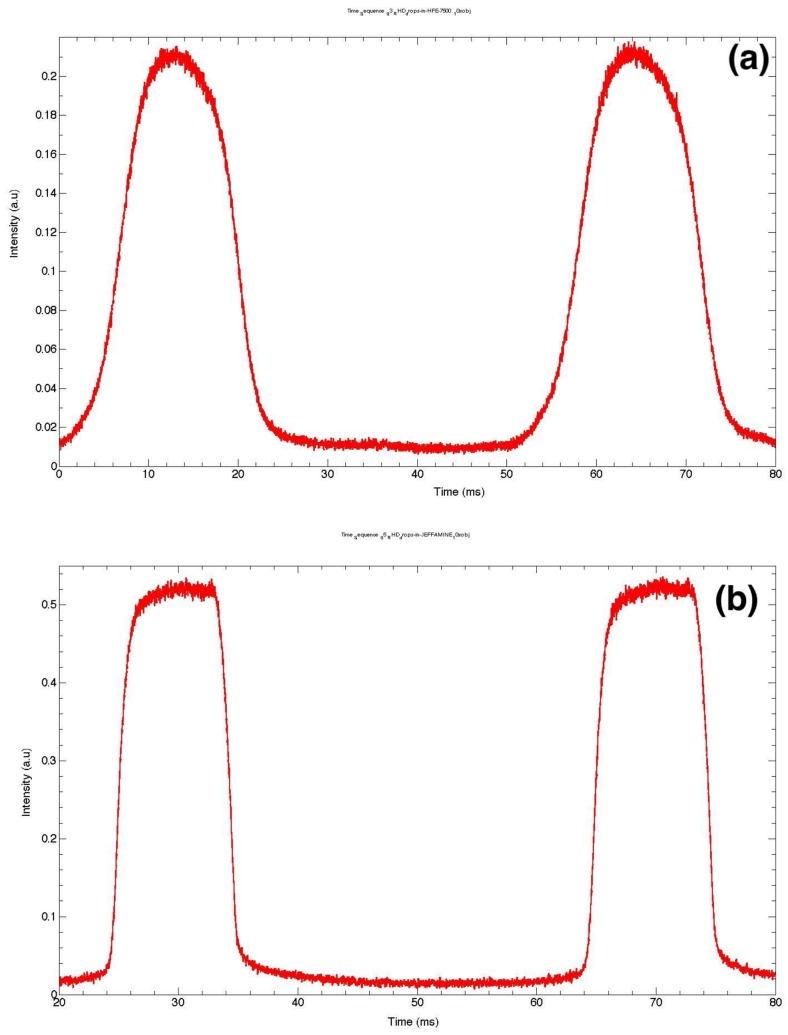

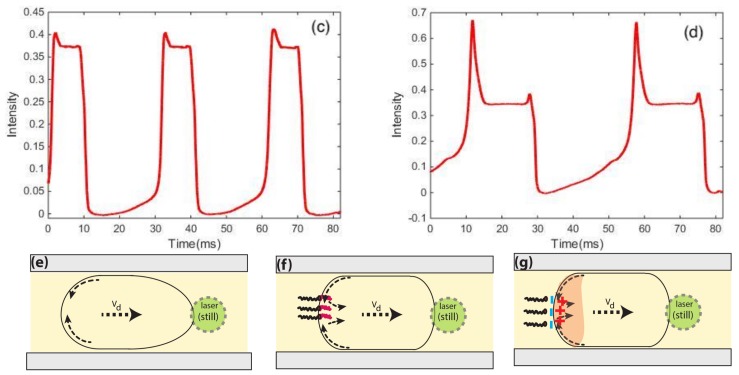
Fluorescence signal from microdroplets (**a**) in HFE7500–without surfactant; (**b**) with surfactant KryJeffa; (**c**) droplets stabilized in Krytox (size around 125 μm); (**d**) big droplets with surfactant Krytox (size above 250 μm); (**e**) plug-like deformation of a large droplet induced by flow of viscous oil; (**f**) heterogeneous distribution of surfactant at the droplet interface; (**g**) in the case of Krytox the distribution of surfactant and its corresponding interaction to charged rhodamine molecules at the rear of the microdroplet.

Real-time fluorescence acquisition not only provides useful information about droplet size and size distribution, it may also give a deeper insight into molecular organization and interactions within the droplet and its interface. For illustration, new results obtained by the authors are presented in Figure 4. They show fluorescence recordings obtained from large droplets containing a rhodamine B fluorescent dye solution (1 mM) and flowing in a roughly square channel with a cross-sectional area of approximately 110 μm × 120 μm. Three different experimental conditions were investigated, differing only in the presence and the type of the used surfactant: Figure 4a corresponds to a case where no surfactant is added; Figure 4b corresponds to a case where a non-ionic surfactant (KryJeffa) is added; Figure 4c,d both correspond to a case where a negatively-charged surfactant (Krytox) was added but for two different droplet sizes—127 μm and 225 μm, respectively.

The bell shape exhibited by the fluorescence intensity of droplets in Figure 4a indicates that droplets more likely adopt a roughly spherical shape: fluorescence intensity starts to increase slightly as the curved interface of the droplet moves more and more across the (still) laser spot before reaching a maximum value when the overlap between the laser footprint and the droplet is at its maximum. One may also note a slight asymmetry of the fluorescence peaks (maximum intensity is shifted towards the left side of the peak). This shift should be correlated to the well-known difference between the profiles of the front and the back of droplets when moving in a flow, as depicted in Figure 4e. It is worth noting that Baret et al. [109] reported direct evidence of the accumulation of surfactant molecules at the back of droplets almost a decade ago, in the flow of a perfluorinated carrier oil (FC40) and using a home-made fluorescent surfactant, namely Krytox-PEO-fluorescein, where a fluorescein isothiocyanate molecule linked to an amine-terminated polyethylene oxide hydrophilic head group was added to Krytox. Interestingly, this surfactant is fluorescent only when the head group is in an aqueous solution, which enabled these authors to monitor the buildup of the surfactant monolayer at the water/oil interface by the readout of droplet interface fluorescence. In contrast, in our study, the accumulation of the surfactant (which is not fluorescent) was indirectly demonstrated by the accumulation of charged rhodamine molecules at the back of the droplets.

In the presence of a surfactant, the recirculation flow induced by the motion of the droplet in the viscous carrier oil generates a heterogeneous distribution of the surfactant at the droplet interface: the surfactant interfacial density becomes higher at the rear region of the droplets than at its front. This effect leads to a rigidification of the droplet interface due to the so-called Marangoni effect [106]. This effect can be clearly seen by comparing fluorescence peaks of Figure 4b–d. In this case, the fluorescence intensity increases rapidly as soon as the laser spot starts to overlap with the droplet front (right region of the fluorescence peaks) and remains almost constant during the flight time τ of the droplet. Hence, in the presence of a surfactant, the droplet deforms less in the flow and is more likely to adopt a plug-like form, as sketched in Figure 4f,g.

In the presence of the negatively-charged Krytox surfactant (Figure 4c,d), one observes a burst of fluorescence at the rear region of the droplet, which corresponds to the left side of the fluorescence peak. By comparing the profiles of the fluorescence peaks obtained with non-ionic surfactant (kryJeffa) and ionic surfactant, the observed burst of fluorescence should be correlated to an increase of the density of the positively-charged fluorescent rhodamine molecules at the rear part of the droplet, which is itself induced by the asymmetric distribution of the surfactant at the droplet interface (see Figure 4f,g).

These results show that the interaction between the droplet content and the surfactant can be detected in a highly sensitive and quantitative manner.

## 5. Ultra-High-Throughput Droplets Production and Detection Methods

Droplets may be produced at kHz rates. Nevertheless, a high acquisition of droplet generation frequency includes droplet monitoring and counting operations based on the detection of optical or electrical signals. Reliable counting and sorting play an important role; similarly, the size distribution of the generated population is important for many applications. Several studies have reported the generation of higher-order droplet frequencies, but the state-of-the-art is limited by the acquisition required to monitor in real-time the generation and rate at which the pace is kept. Throughput achieved up to hundreds of thousands of droplets per second could provide in-depth information about analytes but would require hours of active investigation.

**Figure 5:**
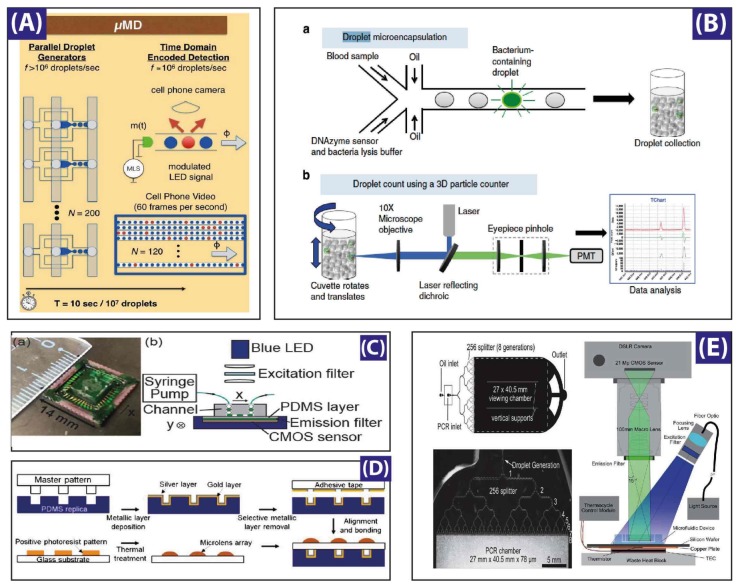
Different studies for droplet generation monitoring: (**A**) A microdroplet megascale detector (μMD) containing a micro-controller-based light emitting diode, a microfluidic device with 120 parallel dropmakers, a cell phone camera for recording, and an off-site data processor. Reproduced with permission from [39]; (**B**) Experimental setup of the Integrated Comprehensive Droplet Digital Detection (IC 3D) system, housing 496 nm and 532 nm laser sources, a dual source single detector scheme modified for the typical experimental needs. A software controlled micro-cuvette holder and rotation unit (1–1100 rpm in rotational speed while 1–15 mm/s vertical translational speed). Reproduced with permission from [36]; (**C**) A CMOS (complementary metal oxide semiconductor)-based sensor with channel bed as closest perimeter for fluorescence detection. The compact sensor assembly consists of a 1280 × 1024 pixel platform, a spin-coated pigment-based band-pass filter, a 250 mW blue LED, and another filter to band limit the light between 457 nm to 492 nm. Reproduced with permission from [34]; (**D**) Integrated micro-optical system with micro-lens assemblies on top of droplet chambers while metallic surfaces at other side of chamber provide optical resonance for improved signal. Reproduced with permission from [38]; (**E**) Experimental stage for digital polymerase chain reaction (PCR) housing a 1 × 256 droplet splitting microfluidic chip on a thermistor stage for PCR thermocycling, a wide field light source, and a digital camera with large field-of-view lens assembly. Reproduced with permission from [37].

To increase both the throughput and the detection limit, many groups have developed improved techniques. Some of the fastest and most reliable methods for ultra-high-throughput utilize, for instance, a laser-induced fluorescence (LIF)-based microfluidic hemocytometer for counting cells encapsulated inside droplets with an average rate of ~600 Hz [130], or amplitude modulation of the acquired signal by a lens-free detection with a rate of 1.7 kHz [33], while a CMOS mounted sensor on a PDMS slab reached a rate of 250 kHz Figure 5C [34]. A high-speed camera assisted by a Fresnel lens reached a read out of 200 kHz [35]. A technique, named “IC 3D” (Integrated Comprehensive Droplet Digital Detection), achieved 100 kHz throughput detection of droplet-encapsulated Blood-DNA, by rotation of a cuvette containing the microdroplets [36] (see system overview in Figure 5B). Another technique where a commercially-available DSLR camera-based system (Figure 5E) developed and recorded at a rate of 250,00 droplets per second [37], while a micro-lens (Figure 5D) array network gathered a read out of 50 kHz [38], and more recently a handy cell phone camera-based read out of one million drops (see Figure 5A) marked the highest data read out and sequencing in digital assays [39].

Another interesting study, reported recently by Shivhare et al. [47] claims the development of a new cost-effective optofluidic dye-free method allowing for a real-time measure of the mean droplet size of a population of droplets and for the measure of droplet size distribution, which is based on the detection of the forward scattered signal (FSC) of an incident non-focused IR laser beam by flowing droplets in the microchannel and the measure of the residence time of these droplets across the incident laser beam. The used microfluidic device consisted of a main drive channel neighboring two control channels, named as grooves. One groove is dedicated to the input laser signal by means of an optical fiber guide, and the other groove is dedicated to the detection part. When a droplet traverses the detection region, it obstructs the passage of the laser beam, which results in a pulse in the detected signal. Shivhare et al. reported a mean droplet size of 15 μm with approximately 10% discrepancy regarding results obtained from optical image analysis. They postulated that the normalized residence time of the generated droplets along the channel is a better measure of the effective droplet size than forward scattered signal, which is correlated nonlinearly to the droplet size [47]. It is interesting to note that for large droplets, similar to our results shown in Figure 4c,d, Shivhare et al. also report a higher scattered signal from the back of the droplet. This result should also be interpreted as the consequence of a greater rigidification of the back of the droplets induced by the addition of the droplet stabilizing surfactant (Span 85) and the recirculation flow, as a rigid interface should scatter more light than a softer interface.

## 6. Optically-Assisted Slicing and Merging of Microdroplets

**Figure 6:**
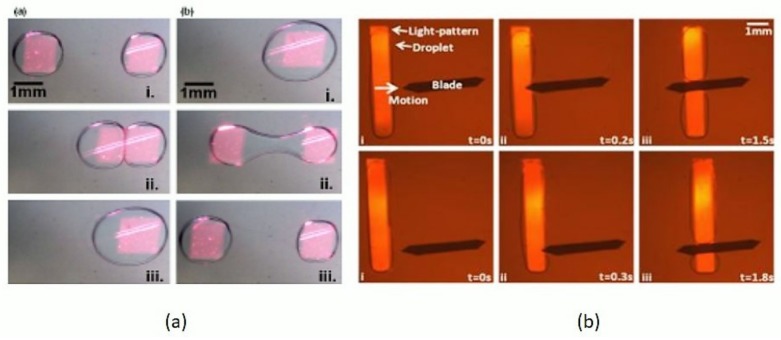
Optical droplets merging and sorting: (**a**) Square-shaped projection patterns moving two droplets towards each other, merged, and sliced back to acquire two droplets. Reproduced with permission from [135]; (**b**) Time sequences of an elongated droplet sliced by on-chip Teflon blade, droplet motion assisted by line projection. Reproduced with permission from [136].

In studies involving bio-molecular assays, the splitting and sorting of microdroplets is a necessity for on-demand droplet size reduction, scale dilution, and volume control of daughter droplets. Splitting can be performed by active methods such as electric field splitting, acoustic, or electro-wetting. Passive splitting could be performed by mechanical in-channel deforming geometries which squeeze and cut the droplet into two. For the passive type, studies have used rectangular and cylindrical channel structures which employ two different types of fabrication modalities. Opto-electrowetting (OEW) [131,132,133,134,135,136,137,138,139] is a novel technique first reported by Chiou et al. [131], where a photoconductive layer is deposited above the electrodes commonly used in EWOD (electro-wetting over dielectric) [140,141]. The working principle involves a local change in the surface properties (contact angle and surface tension at light-spot) between the conductive layers and the liquid of interest. The use of this kind of OEW device for the optical manipulation of droplets by virtual electrodes [132,134,135,136,137,138,139], or by local change of the hydrophobicity of the radiated surface provides significant change in the local surface tension; thus, droplets can be moved, merged, patterned, and diffused. The all-optical elemental control involves light-assisted digital microfluidic chips (LADM) [135,136,137,138] for droplet movement by a laser source [131,132,133,134] or by data projectors [135,136,137,138,139]. Pei et al. [135] use data projection to move, merge, elongate, and divide microdroplets on the surface of a dielectric layer by utilizing a multi-pattern projection method, thus resulting in a unique multiple drop generation, movement, and control (Figure 6a). Later, Pei et al. [136] demonstrated a new approach by introducing a system of Teflon blades (Figure 6b) between two electrodes for slicing microdroplets at the nL scale, and the splitting ratio was reported to be between 10% and 90%.

**Figure 7:**
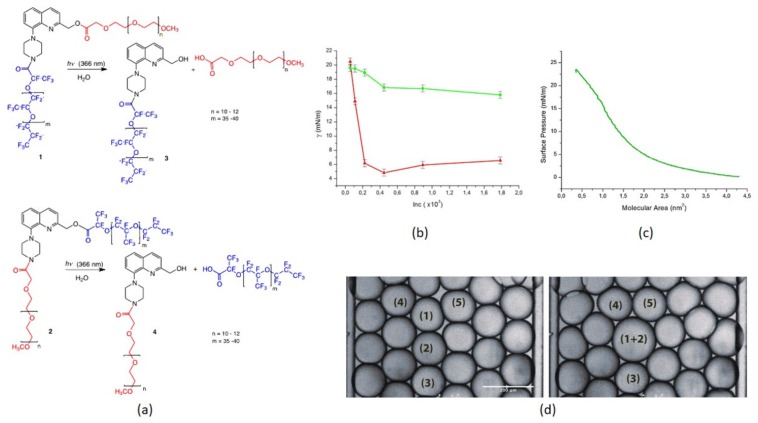
Surfactants 1 and 2: (**a**) Chemical composition; (**b**) Interfacial tension versus concentration, red curve for Surfactant 1 and green curve for Surfactant 2; (**c**) Surface pressure versus molecular area; (**d**) Light-induced merging of droplets 1 and 2. Reproduced with permission from [32].

To add a new study, the breakage of long caged-group molecules grafted on the droplet periphery is a novel idea we reported in a recent work [32]. The technique involves the photolysis of a photosensitive surfactant chain. A pulsed laser source at 1 kHz depletes the surfactant monolayer grafted on the droplet interface. Two surfactant compounds were fabricated with 8-piperazinyl-2-hydroxymethyl-quinoline (8-PHQ), named surfactant 1 and 2 (see Figure 7). Surfactant 1 resulted in stable monodisperse droplet formation and controlled release. Various concentrations resulted in significant reductions in time of merging up to ~2 s, while the second compound (Surfactant 2) was unable to perform the controlled merging of microdroplets. Pendent drop tensiometry (Figure 7b) and Langmuir monolayer method (Figure 7c) were used to evaluate interfacial tension versus concentration and corresponding molecular area occupied by a single molecule under the compression of monolayers. Figure 7d shows the time lapse for the merging of two monodisperse droplets. These methods could develop a new approach for medical diagnostics and treatment for on-the-spot on-demand region-selective target, release, and treat methodology.

## 7. Microdroplets as Optical Probes for 3D Imaging and Sensing

The production of a large population of highly monodisperse droplets with highly controllable optical features gave rise to very interesting applications in the field of 3D imaging and sensing, either as a tunable liquid double emulsion [63], solid spherical particles [55,142], or soft reconfigurable core with elastic shell microparticles [56]. The periphery of the spherical microdroplets performs the convergence or divergence of the incident light provided that the morphology or fabrication protocols are addressed properly. Besides the use of single micro-optical lens systems for imaging and improved resolution, arrayed networks increase spatial resolution and fluorescence signal detection many times over. For instance, Lim et al. [38] reported an eight-times increase in the fluorescence signal by introducing a soft micro-lenses system on the top of metallic coated micro-channels. Additionally, Ghenuche et al. [142] reported on another arrayed micro-optical lenses system based on microspheres, which enables the parallel detection of single fluorescent molecules in a multi-focus nanojet experimental technique. Ghenuche et al. utilized latex micro-spheres of size 2 μm for the generation of such nanojets. In order to perform fluorescence correlation spectroscopy by nanojets, they illuminated a 10 μm spot (approximately 25 micro-spheres) by a low numerical aperture (NA) objective and acquired sensitivity as low as 20 pico-molar fluorescent dye concentration. These type of detection schemes assisted by micro-optical elements yield good estimations of concentration (in the pM range), diffusion coefficient, and relative hydrodynamic radius of the dye molecules.

**Figure 8:**
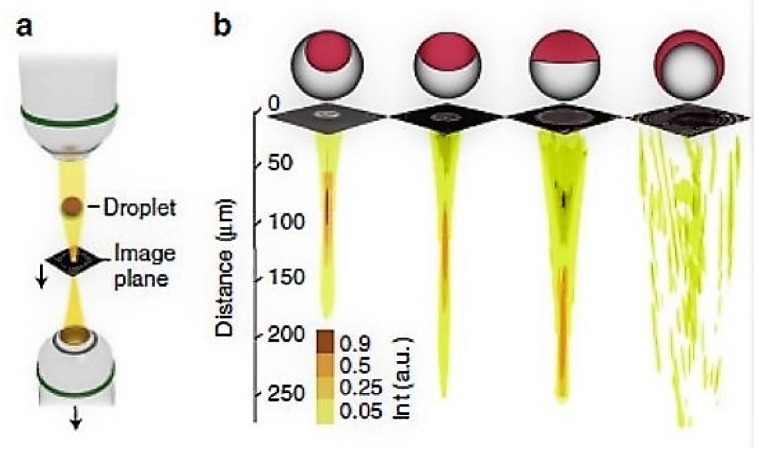
Imaging using microdroplets: setup for light input from the top with an adjustable image plane, droplets on a substrate, image plane, and bottom collection objective with vertical translation, characteristic focal length tunability based on the morphology of the emulsion, and corresponding fully converging-to-fully diverging mechanism. Reproduced with permission from [63].

Another interesting study reported by Nagelberg et al. [63], who used microdroplets as optical microlenses with a tunable focal length based on the concentration variations of the surfactant and the drop–disperse–drop double emulsion phases. They reported the use of hydrocarbons–fluorocarbon–aqueous phases for focal length tunability by adjusting the relationship among the refractive indexes of the interfaces involved, be it the denser fluid inside causing a converging lens or be the denser fluid in the drop shell resulting in a diverging lens system. Figure 8 represents the different types of used droplets, from janus drops to double emulsions and inverse emulsions. The demand for this kind of optical tunability finds potential in super-resolution imaging and displays, light field displays, liquid crystal displays, digital micro-mirror displays, optical tweezers, and medical diagnostic and investigation probes.

## 8. Conclusions

This review outlines recent advances in droplet optofluidics and focuses more particularly on analysis tools for producing highly monodisperse droplets using microfluidic devices and optical methods at high throughput. The optical qualities of micro-droplets and their high potential for applications in biology and chemistry open prospects for applications in drop/capsule/container-on-demand, lab-on-a-chip, cellular matrix mimicking, reconfigurable drug carriers, in-channel processes, and incubation and surface modification in particular as development tools for highly sensitive sensors. We first present the basic concepts of droplet microfluidics, which include microfluidic devices, droplets fabrication, and stabilization. Particular attention is given to experimental optical methods developed for a real-time measurement of droplet size and size distribution, since light provides flexibility and wavelength/intensity tunability. Among the developed optical methods, real-time fluorescence measurement is a highly sensitive one. It allows not only for the detection and analysis of the size and content of droplets, but also for a deep insight into the molecular interaction between droplet contents and the surrounding surfactant molecules. We also present an extension of droplet optofluidics which uses microdroplets as reconfigurable micro-lenses. In summary, droplet microfluidics joined by the potential of optics as a probing and extracting method could open up new dimensions to biomimetic reconstruction and optical control on one hand and smart drug delivery optofluidics micro-systems on the other.

## Abbreviations

The following abbreviations are used in this manuscript:

DNA	Deoxyribonucleic acid
SAXS	small angle X-ray scattering
PDMS	Polydimethylsiloxane
ILIDS	interferometric laser imaging for droplet sizing
CMOS	Complementary metal–oxide–semiconductor
IC 3D	integrated comprehensive droplet digital detection
DSLR	Digital single-lens reflex camera
μMD	Microdroplet megascale detector
PCR	Polymerase chain reaction
LIF	Laser-induced fluorescence
PEG	Polyethylene glycol
EWOD	Electrowetting over dielectric
OEW	Opto-electrowetting
PS	Polystyrene
PMMA	Poly-methyl-methacrylate
PC	Polycarbonate
FSC	Forward scattered signal
LADM	Light assisted digital microfluidic chip
W/O	Water-in-oil emulsion
O/W	Oil-in-water emulsion
pH	Potential of hydrogen
